# Quantifying the Magnitude and Longevity of the Effect of Repetitive Head Impacts in Adolescent Soccer Players: Deleterious Effect of Long Headers Extend Beyond a Month

**DOI:** 10.1089/neur.2022.0085

**Published:** 2023-04-21

**Authors:** Farzin Shamloo, Maria Kon, Elizabeth Ritter, Anne B. Sereno

**Affiliations:** ^1^ Department of Psychological Sciences, Purdue University, West Lafayette, Indiana, USA.; ^2^ University of North Carolina Health, Chapel Hill, North Carolina, USA.; ^3^ Department of Neurobiology and Anatomy, McGovern Medical School, University of Texas Health Science Center at Houston, Houston, Texas, USA.; ^4^ Weldon School of Biomedical Engineering, Purdue University, West Lafayette, Indiana, USA.; ^5^ Navy Center for Applied Research in Artificial Intelligence, Naval Research Laboratory, Washington, DC, USA.

**Keywords:** anti-point task, Bayesian statistics, repetitive head impacts, pro-point task, soccer

## Abstract

There is growing interest in the effects of sports-related repetitive head impacts (RHIs) on athletes' cognitive capabilities. This study examines the effect of RHIs in data collected from adolescent athletes to estimate the magnitude and longevity of RHIs on sensorimotor and cognitive performance. A non-linear regression model estimated the longevity of RHI effects by adding a half-life parameter embedded in an exponential decay function. A model estimate of this parameter allows the possibility of RHI effects to attenuate over time and introduces a mechanism to study the cumulative effect of RHIs. The posterior distribution of the half-life parameter associated with short-distance headers (<30 m) is centered around 6 days, whereas the posterior distribution of the half-life parameter associated with long-distance headers extends beyond a month. Additionally, the magnitude of the effect of each short header is around 3 times smaller than that of a long header. The results indicate that, on both tasks, response time (RT) changes after long headers are bigger in magnitude and last longer compared to the effects of short headers. Most important, we demonstrate that deleterious effects of long headers extend beyond 1 month. Although estimates are based on data from a relatively short-duration study with a relatively small sample size, the proposed model provides a mechanism to estimate long-term behavioral slowing from RHIs, which may be helpful to reduce the risk of additional injury. Finally, differences in the longevity of the effects of short and long RHIs may help to explain the large variance found between biomechanical input and clinical outcome in studies of concussion tolerance.

## Introduction

There is growing interest in the effects of sports-related repetitive head impacts (RHIs) on athletes' cognitive capabilities. In a retrospective analysis, Mackay and colleagues^1^ showed that the mortality rate attributable to neurodegenerative disease was higher in former professional soccer players compared to matched controls. Recent evidence shows associations between RHIs and various neurological and behavioral measures: default mode network changes^2,3^; memory deficits^4–6^; white matter alterations^7–11^; neurometabolic alterations^12,13^; and neurological impairments.^13–15^ Despite the success of imaging techniques in linking subconcussive impacts to brain alterations, there are limitations (cost, feasibility) of such tools for regular, repeated testing. Although behavioral measures offer alternatives, it is challenging to find one sufficiently sensitive to RHIs. Sereno and colleagues^16^ introduced a touch-based method for accurately recording response times (RTs) to touchscreens that can detect behavioral changes related to RHIs,^17–19^ where previous studies failed to do so.^20,21^ Here, we aim to estimate the magnitude and longevity of the effect of RHIs on athletes' RT by applying a Bayesian model to data from a study that used a pointing task.^18^

Quantifying the magnitude and longevity of RHI effects provides a way of estimating the cumulative effect of RHIs, advancing theory, and aiding clinical practice. For example, cumulative effects may explain the variability between biomechanical input and clinical outcome across persons.^22^ Likewise, if RT is substantially slowed down because of accumulated RHIs, this RT slowing can reduce an athlete's ability to avoid additional contacts and increase injury^23,24^ and concussion risk.^25^ Thus, being able to quantify the accumulated effect of different RHIs may help to identify concussion risk and aid clinical practice.

### Temporal aspects of head impacts

Various assumptions and methods have been used to account for the cumulative aspect of RHIs. Some studies divide the experiment into various temporal stages and associate the output measure to each of the pre-defined stages,^4^ whereas others count all headers over a time period preceding the testing session.^7,26^ Another approach is to calculate the average peak translational acceleration of head impacts above a set threshold before a testing session.^12^ Such approaches, however, do not take into account the temporal proximity of head impacts to the testing session. For example, an impact that happened 3 days before testing is assumed to have the same effect as an impact that happened 10 days before testing. To date, most studies focus on cumulative effects over a period of weeks and months without consideration of the timing of impacts with respect to output measures. In this study, a half-life parameter is introduced. Generally, half-life is a characteristic of an exponential decay function and is defined as the time required for a quantity to decay to half of its initial value. For this data set, the half-life parameter allows the possibility of the impacts' effect on behavioral outcome (i.e., RT) to attenuate over time, thus allowing us to examine the temporal aspects of RHIs on behavior (see Supplementary Appendix C, Equations A.2 and A.3).

We apply a Bayesian model to data reported in Koerte and colleagues^18^ and quantify the magnitude and longevity of the effects of short-distance (<30 m) and long-distance (>30 m) RHIs. Longevity of the effect of RHIs is estimated by adding a half-life parameter for short and long headers. Using the Bayesian method, we will get the distribution of the magnitude and longevity of each type of RHI for each task, giving us a more complete picture of the effects of RHIs and allowing us to better understand the consequences of these impacts.

## Methods

We analyzed fully deidentified data from Koerte and colleagues.^18^ Key details are summarized in the next two sections (see Koerte and colleagues^18^ for additional details).

### Participants

Sixteen male soccer players (mean age, 15.7 ± 0.7 years) and a comparison group of 14 male non-contact athletes (mean age, 14.9 ± 1.1 years) recruited from competitive athletic clubs in Germany participated in the study (ages 13–19). Athletes diagnosed with (or suspected to have) a TBI, as defined professionally according to the international consensus statement,^27^ within a year and athletes with past clinically defined neurological, psychiatric, or learning disorders were excluded.

### Procedure

#### Design

The design was a prospective, longitudinal, observational, comparison study between soccer and non-contact athletes during training session days in the spring off-season. A typical training consisted of a mix of drills and scrimmages. Athletes were tested twice in each training day, once before training (pre-session) and once within 15 min after training (post-session). Athletes completed 1–4 days of testing per week. The experimenter collected data for each participant who attended practice that day (number of testing sessions: mean (*M*) = 7.8, standard deviation (*SD*) = 2.6 for soccer players; *M* = 4.2, *SD* = 2.7 for non-contact). A detailed examination and plots of time-related factors of testing are provided in Supplementary Appendix C.

#### Exposure to repetitive head impacts

Soccer players were exposed to RHIs during training on testing days (*M* = 6.5, *median* = 5). A trained research assistant counted and classified headers of each soccer player through observation. Headers were classified into three types: “short” (e.g., short-range headers in a practice drill); “long” (e.g., headers resulting from a goalkeeper or corner kick, >30 m); and “high” (e.g., headers after a goalkeeper punt). Because previous work suggests that velocity is a factor determining head impact severity^28,29^ and because velocities of long and high headers are similar, we reclassified high headers as long headers. Athletes in the non-contact group did not experience any head impacts.

#### Stimulus and tasks

**FIG. 1.**
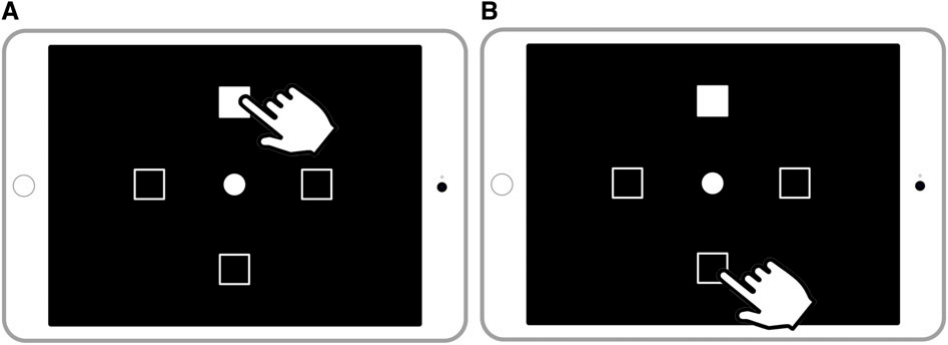
Schematic of the two tasks performed by athletes. Subjects were instructed to place and hold their index finger on the circle at the center of the iPad and tap the goal location (correct goal location indicated by the white index finger cursor in the figure) as soon as the stimulus (white square) would appear. In the pro-point task (**A**), the goal location was the location where the stimulus appeared, and in the anti-point task (**B**), the goal location was the square on the opposite side of where the stimulus appeared.

Two tasks were performed on tablets: the pro-point and anti-point tasks, touch-based versions of pro-saccade and anti-saccade tasks used to measure sensorimotor and executive function, respectively.^30^ In both tasks, athletes were asked to place and hold their index finger on a circle in the tablet center and wait until a stimulus appeared. The stimulus (a white square) appeared in one of four possible locations. In the pro-point task, subjects were instructed to tap the square where the stimulus appeared as quickly as possible (Fig. 1A), whereas in the anti-point task, they tapped the square opposite the stimulus (Fig. 1B).

Each task was presented in a block with at least 48 trials (incorrect trials were repeated). RT, the duration from onset of the stimulus to the tap of the goal location,^31^ was measured. After filtering out all the incorrect trials (3.6%), the “outliers” package^32^ for R was used to perform a chi-squared test for outlier detection,^33^ and outlier trials (4.2% of correct trials) were excluded from further analysis.

### Statistical analysis

A non-linear hierarchical regression model was used to quantify effects of short and long headers on RT in pro-point and anti-point tasks while allowing the possibility of the impacts' effect to attenuate over time by adding a half-life parameter embedded in an exponential decay function. The model uses one parameter to estimate the magnitude of the effect of RHIs for each head impact type (with coefficients for long and short headers denoted by $\beta_{Short}$ and $\beta_{Long}$, respectively) and one parameter for estimating the longevity of the effect of RHIs for head impact type (with coefficients for long and short headers denoted by $hl_{S}$ and $hl_{L}$, respectively).

In addition to these main parameters of interest, the model also estimates the effect of additional time-related factors of testing, including control for the number of previous sessions (to control for practice effects), days since the first session (to control for developmental changes^34^), days since the previous session (to control for any carryover effects), and age of the athletes. Model description and a detailed explanation of the construction of covariates and parameters are provided in Supplementary Appendix A. The parameters of the model were estimated using Bayesian methods,^35,36^ and key statistics for all the population level parameters are provided. Additionally, for parameters that are the focus of this study, the posterior distributions (i.e., the probability distribution of the parameters conditioned on the observed data) of these parameters are shown in order to better understand and carefully examine those parameters. An explanation of the inference method is given in Supplementary Appendix B.

## Results

Table 1 lists, for each population-level parameter, the posterior estimate and 95% credible interval. Intercept estimates for pro-point and anti-point were 450.64 and 540.43 ms, respectively. To make interpretation easier, the effects of other parameters are visualized in subsequent sections. For a more detailed version of Table 1, with all other population-level parameters, see Supplementary Appendix A.

**Table 1.** Posterior Estimate and 95% Credible Intervals for Population-Level Parameters Related to the Magnitude and Longevity of the head impacts

		Label	Estimate (mean, ms)	95% credible interval (ms)
$\beta_{1}$	Pro-point	Intercept	450.64	(430.42, 471.46)
	Anti-point		540.43	(518.67, 562.87)
$\beta_{Short}$	Pro-point	Short headers	–1.10	(−1.31, −0.91)
	Anti-point		–1.17	(−1.39, −0.95)
$hl_{S}$	Pro-point	Half-life (short headers)	5.19	(4.27, 6.09)
	Anti-point		6.51	(5.02, 8.15)
$\beta_{Long}$	Pro-point	Long headers	3.69	(3.20, 4.17)
	Anti-point		3.20	(2.64, 3.77)
$hl_{L}$	Pro-point	Half-life (long headers)	309.07	(13.76, 491.01)
	Anti-point		358.08	(100.49, 494.09)

### Longevity of short and long headers

**FIG. 2.**
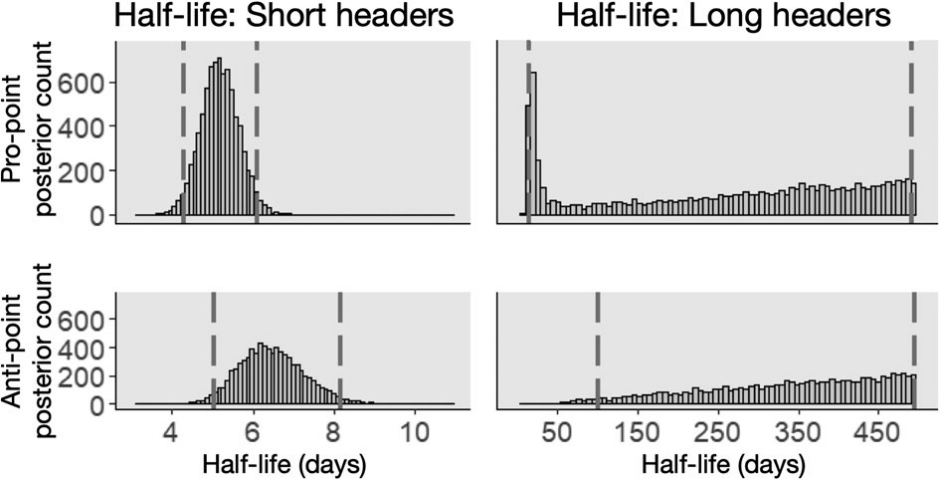
Histograms of the half-life of the short and long heading effect in soccer players. Short headers have a short half-life (around a week) for both tasks, but long headers affect the two tasks differently. The effect of long headers on the pro-point task has a peak of around 14 days, but then a very long tail (which pulls the mean of samples to 309 days). On the other hand, the posterior distribution of the half-life of long headers on the anti-point task is almost uniform, between 150 and 500 (with a mean of 358 days), which means almost no attenuation (given that the longest gap between a header and a testing session was 29 days). Dashed vertical lines show the 95% credible interval.

Figure 2 shows the posterior distribution for the half-life of short and long headers for pro-point (top row) and anti-point (bottom row) tasks. Short headers had a relatively short half-life in both tasks (around 4 and 8 days), whereas long headers had a longer half-life and different distributional characteristics for the two tasks. The effect of long headers on the pro-point task was characterized by a distribution that peaked around 14 days, but had a heavy tail that pulled the mean of the half-life to 309 days. On the other hand, the distribution of the effect of long headers on the anti-point task had a mean of 358 days and was almost flat between 150 and 500 days (where 500 days is the upper limit of the sampler). Given that the biggest gap between a header and a testing session is 29 days, the posterior samples of the half-life of long headers on the anti-point task suggest that there was no attenuation for any of the long headers in this study.

The half-life was constrained to be smaller than 500 days in order to prevent the sampler from seeking bigger $hl_{S}$ and $hl_{L}$ values and not converging (in case of no attenuation), and Figure 2 (bottom right) shows the samples stacked up before the imposed constraint. However, this conclusion is limited by the data we examined. For example, if the true half-life of the effect of long headers on anti-point were much larger than the time period we examined, such as 150 days, our study with only 29 days between the first and last sessions for soccer players would not have provided the data to detect such an attenuation, whereas a study across 300 days would have been able to detect the true half-life. The sampler's inability to peak anywhere between 150 and 500 days and showing an almost uniform characteristic reflects this limitation in time span, which would change in a data set with a wider span.

### Magnitude of the effect of headers

**FIG. 3.**
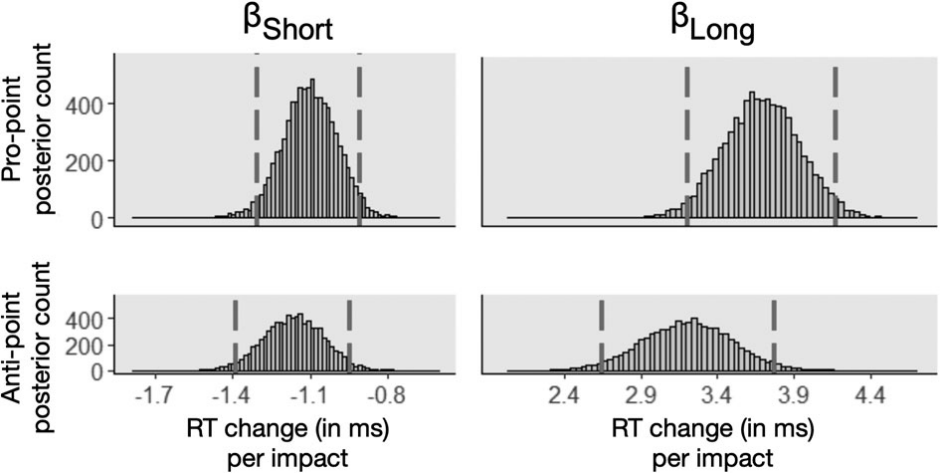
Histogram of header-related coefficients. Short headers are correlated with smaller magnitude and faster RTs, and long headers are correlated with larger magnitude and slower RTs. Dashed vertical lines show the 95% credible interval, and RT indicates response time.

Figure 3 illustrates the posterior distribution of the header-related regression coefficients ($\beta_{Short},\beta_{Long}$; see Supplementary Appendix B for a general discussion on posterior distributions). Top and bottom rows show the pro-point and anti-point tasks, respectively, and illustrate that the header-related coefficient is negative for short headers and positive for long headers in both tasks. Overall, the results of this and previous sections suggest that short headers have a short-lived, small positive effect on RT (making participants faster on both tasks) and long headers have a longer-lasting, larger negative effect on RT (making participants slower on both tasks).

### Time-related factors of testing

There were significant effects of practice, developmental changes, and carryover effects, such that 1) more practice sessions on the task (more so for anti-point), 2) longer experimental time intervals examined, and 3) proximity to previous testing sessions all resulted in greater RT benefits. All three factors impacted RT: 1) participants improved an average of 6.3 ms per session (5.21 ms pro-point, 7.34 ms anti-point); 2) the biggest possible developmental-related change occurred in the control group and was 91 days or an approximate 30.94-ms decrease in RT; and, finally, 3) the results show a carryover effect from previous testing sessions that attenuates relatively fast on the pro-point task (half-life of ∼4 days), but attenuates much slower for anti-point (half-life ∼40 days). The positive carryover effect can be caused by physical exercise done in the previous session or by a recency effect of doing a task again. Previous research suggests that physical exercise can result in immediate, longer-term changes in the brain^37–39^ and can positively affect cognitive abilities of healthy young adults.^40–42^ Therefore, it is likely that some of the carryover effect was caused by physical exercise. The posterior estimates, 95% credible intervals, and other statistics are provided in Table A.1 of Supplementary Appendix A. A more detailed examination of the results of time-related factors of testing is provided in Supplementary Appendix C.

## Discussion

Using a Bayesian approach, we examined RT data from adolescent athletes with and without exposure to RHIs to better understand the magnitude and longevity of the effects of short and long headers. For short headers, half-life estimates for pro-point and anti-point tasks were similar and short (5.19 and 6.51 days, respectively), suggesting that effects of short headers do not last long. However, this was not the case for long headers. The effect of long headers was relatively shorter on pro-point (mode, 14 days; mean, 309 days) compared to anti-point (flat distribution with a mean of 358 days and samples stacked up before an imposed upper limit). In addition to longer-lasting effects of long headers compared to short headers, long headers also had a bigger impact on RT compared to short headers (∼3 times larger). Thus, whereas the small-magnitude, short-lived effects of short headers were positive on RT, the larger and longer-lasting effects of long headers cumulated and slowed down athletes' RT.

To aid in the interpretation of the effects of short and long headers, an example can help illustrate how magnitude and longevity are simultaneously taken into account in the model, according to Equations A.1 and A.4 of Supplementary Appendix A. For example, consider a hypothetical athlete with 36 short and 6 long headers (approximately group averages) before the fifth session. Taking 3.2 and −1.17 ms as the estimates for $\beta_{Long}$ and $\beta_{Short}$ (Table 1) and assuming the gap between fifth session and previous four sessions to be close to group average (approximately 3, 5, 7, and 8 days), the effect of accumulated short and long headers would be −18.68 and 20.68 ms, respectively. Note that given the short half-life of short headers (half-life around a week), the effects of short headers would go down to zero very fast whereas the effect of long headers would last much longer. For example, adding 10 days to the calculated gaps of the mentioned example and assuming no additional RHIs, the effect of short headers would virtually disappear (−0.48 ms), whereas the effect of long headers would barely drop (20.66 ms).

Much earlier behavioral work in humans has not been able to detect reliable changes with concussion.^43,44^ Despite reliable behavioral changes after RHIs documented in animal models,^45^ results have been mixed in human studies, with some suggesting that sports-related RHIs result in little or no postural, behavioral, or cognitive deficits,^46–50^ whereas others indicate that these RHIs do result in significant changes in posture, behavior, or cognition.^17,19,51–53^ Here, we are able to estimate, in adolescent male athletes, the magnitude and longevity of the effect of RHIs on RT and show that some deleterious effects of long headers on executive function do not attenuate over the period we observed (∼1 month). Quantifying the magnitude and longevity of the effect of different RHIs is critical for advancing understanding and practice. The long-lasting effect of long headers may help explain the variability observed in the relation between the magnitude of a given head impact and clinical response across the study subjects.^22^ Further, estimates of the magnitude and longevity of the effect of different RHIs may aid in clinical management. As we show, only long RHIs have long-term (and larger) deleterious effects. Further, these long-term effects may increase the risk of concussion, by slowing down the cognitive function of athletes and lowering their ability to avoid damaging contact.^23,24^

Tracking athletes' head impacts during a season, which may be more effectively tracked using wearable head impact sensors, and calculating RT accumulations and slowing may help to identify vulnerable athletes and reduce concussion risk. These findings may also suggest that long headers should be trained less to avoid the accumulation of potentially harmful effects.

Our results also show the benefits of previous practice, smaller gaps between successive sessions, as well as developmental changes. It was important to reformulate these time-related factors of testing and include them in the model given that there is not always a perfect alignment between contact and non-contact or control groups. For example, not taking into account soccer players having smaller gaps between successive sessions compared to the control group results in a slight underestimate of the negative effect of head impacts in the soccer players (i.e., the RT benefit of closer spacing of sessions in soccer players would reduce the estimate of the RT cost of heading).

Finally, for this study, there were a number of advantages for using a Bayesian, as opposed to a hypothesis testing or frequentist, approach (see Supplementary Appendix B for additional details). Unlike the frequentist approach, which can only be used to reject a null hypothesis, the Bayesian approach can also provide evidence in support of a null hypothesis, which is especially advantageous in exploratory analyses like this study. Additionally, the Bayesian approach gives the distribution of the parameter that can be used to calculate any probability associated with that parameter, and thus provides much more information than stating whether there is evidence to reject the null hypothesis. Finally, a Bayesian approach does not have the power issue when sample size is small and is better for dealing with small sample sizes in general.^54^

### Limitations and future directions

The Bayesian model is simple and the statistical assumptions minimal (Table A.1, Supplementary Appendix A). The model assumes the effect of RHIs to be cumulative, with the effect of more recent headers being stronger. The short and long headers are not two different covariates, but rather summed together as one variable (i.e., the cumulative effect of RHIs). The distinction between short and long headers (separate parameters) is used to allow and estimate different weights for each type of header, but in the end, all headers preceding a testing session are summed. In addition, the model has one random effect of subject on the RT intercept (Equation A.1), but other parameters do not have a varying subject element to allow for individual differences.

Making a more complex model allowing and formulating individual differences in the RHI parameters (e.g., magnitude or longevity) or additional interactions (e.g., formulating the effect of previous short headers on the magnitude and longevity of a long header and *vice versa*) would require many additional assumptions and parameters to be estimated and may make model convergence problematic with the current data set. Future work with more elaborate designs, separated conditions, or larger data sets would be needed to explore more complex models and interactions. Although a larger sample size would likely have the advantage of tighter parameter estimates, the Bayesian approach used is less susceptible to issues that arise with small sample sizes using frequentist methods, provided that justified, thoughtful priors are used.^54,55^

The data set we analyzed had some limitations in data collection methods.^18^ First, the study only used one observer to classify headers. Lacking multiple observers, reliability across observers could not be calculated. In addition, the observer classified the headers qualitatively. As noted in the original report,^18^ although future studies would benefit from more accurate, quantitative means of classifying headers and specifying their properties such as speed, we are confident that, because of a distinct difference between short and long headers, the data are sufficiently robust and reliable. The study also chose a relatively limited duration (a single season), in part because of the known rapid developmental changes occurring in adolescence.^34^ However, for long headers, the half-life for the anti-point task appeared to extend beyond the period studied and thus limited our ability to observe an attenuation of the effect of long headers and estimate their true half-life. Future work should explore designs with longer durations. Of course, such studies will incur greater developmental changes, but the inclusion of a non-contact athletic control group would allow a relatively accurate estimate of developmental RT benefits. Regarding carryover effects, future studies could be designed to try to tease apart the temporal effects of exercise benefits from any effects of recency in repeated testing, such as testing on days with no training.

In this study, we analyzed data from high-performance male athletes, and it is possible that conclusions may not be similar for female athletes. Also, the number of RHIs depends on various factors such as type of the sport, level of play, position, etc.^56^ For these reasons, there is the risk to simply generalize the results of one group to all the others. Finally, the study examined a specific type of RHI (i.e., heading the ball in soccer), and the findings may not generalize to other types of RHIs (e.g., collisions in American football). In order to get a better picture of the magnitude and longevity of impacts, future work should examine data from different sexes, sports, levels of play, or magnitude of impacts.

## Conclusion

We examined the effect of RHIs in adolescent athletes using a Bayesian analysis, including time-related aspects of head impacts and testing. We find that changes in RT after long headers are of greater magnitude and last much longer than short headers, with deleterious effects of long headers on a task of executive function extending beyond a month. Further, we found that RT benefits increase with the number of previous tests, greater temporal intervals (attributable to the rapid developmental changes in adolescence), as well as shorter gaps between testing sessions. Quantifying the magnitude and longevity of the effect of different RHIs is critical for both advancing our theoretical understanding as well as providing approaches to improve clinical practice.

## Transparency, Rigor, and Reproducibility Summary

All experiments were in accordance with the Declaration of Helsinki and approved by the institutional ethics committees of the University of Texas Health Science Center and Purdue University. Written informed consent was obtained from each participant as well as their legal guardian if the participant's age was below 18 years. Posterior samples of the population-level parameters are available upon request. The code is available upon request.

## Supplementary Material

Supplemental data

Supplemental data

Supplemental data

## Abbreviations Used

*M*: mean
RHIs: repetitive head impacts
RT: response time
*SD*: standard deviation
TBI: traumatic brain injury
