# Supplementary material for: Quantifying the Magnitude and Longevity of the Effect of Repetitive Head Impacts in Adolescent Soccer Players: Deleterious Effect of Long Headers Extend Beyond a Month
Source: Neurotrauma Rep. 2023 Apr 21;4(1):267–75. doi: 10.1089/neur.2022.0085 (PMC10122256; doi:10.1089/neur.2022.0085)
Supplement: Supplemental data [file Suppl_AppendixC.docx]

# Appendix C

# Time related factors of testing

Figure C.1 shows the posterior samples’ histograms of $\beta_{Practice}$ and $\beta_{Days}$) for both tasks. The first row corresponds to the Pro-point task and the second row corresponds to the Anti-point task.


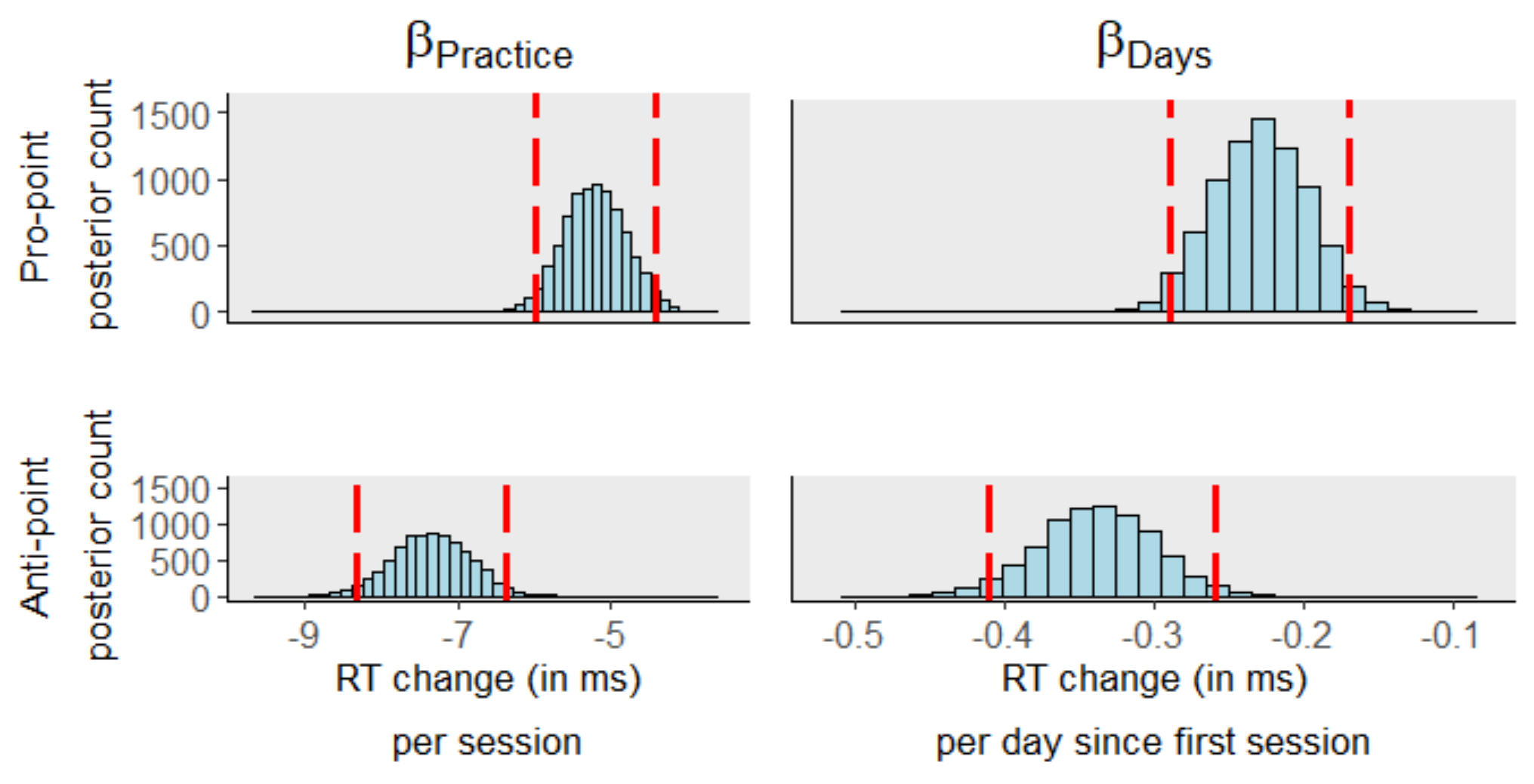


Figure C.1. Histogram of the posterior samples of $\beta_{Practice}$ and $\beta_{Days}$ for both the Pro-point and Anti-point tasks. As the figures show, more practice on the task ($\beta_{Practice}$) makes participants faster in both tasks. In addition, there are also small developmental related reductions in RT ($\beta_{Days}$) of both tasks over the short time interval examined in the present study. The dashed red lines show the 95% credible interval.

The interpretation of $\beta_{Practice}$ is very straightforward: In both tasks, there is clearly a practice effect and it is stronger in the Anti-point task. Each practice session on average makes a participant’s Pro-point and Anti-point RT around 5.21 ms and 7.34 ms faster.

Figure C.2 shows the percentage of sessions with $0, 1, \ldots, 10$ practices for control and soccer group to examine whether the estimated practice factor t_p_ affected soccer group and control group differently. As the figure shows, in the soccer group almost all participants did at least four sessions of testing as suggested by the almost flat blue bars for $t_{p}=0, 1, 2, 3$, with fewer participants having up to 11 testing sessions and therefore there were less sessions with $t_{p}=4, 5, \ldots, 10$. A similar pattern exists for the control group (red bars) where the decrease of percentage of sessions as $t_{p}$ increases also indicates that fewer control participants completed up to eight testing sessions. The histograms for soccer and control group look relatively similar but there is a slight unbalance in the distribution of $t_{p}$across two group with soccer group on average having more practice and thus benefiting more from the RT changes caused by practice.


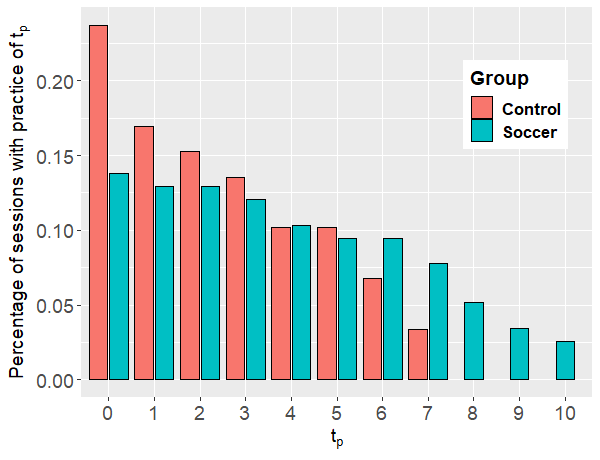


Figure C.2. Bar graph of the percentage of sessions with $0, 1, \ldots, 10$ practices for control and soccer group. The bar graph shows that the covariate $t_{p}$was relatively balanced across groups with a slight unbalance in the two ends of the graph.

The posterior samples of $\beta_{Days}$ for both Pro-point and Anti-point are negative (Figure C.1, right column). The mean of the samples for Pro-point and Anti-point is -0.23 and -0.34 ms respectively, which are the average drop in RT for each day after the first session. Figure C.3 shows the histogram of $t_{d}$ for control and soccer groups and shows that the control group participated in the experiment over a larger time span. Taking -0.34 ms as the estimate for $\beta_{Days}$, the biggest possible developmental decrease in RT for soccer players is about 29 $\times0.34=9.86$ ms and for the control group about $91\times0.34=30.94$ ms. Hence, developmental decreases in RT explain some of the differences between mean RTs in soccer players and the control group.


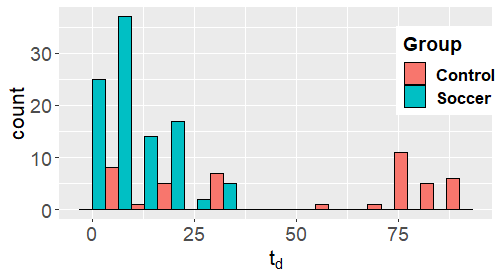


Figure C.3. Histogram of $t_{d}$ for control and soccer groups. The histogram shows that the control group participated in the experiment over a larger time span than did the soccer players (median time span for soccer and control groups are seven and seventy days respectively).

Figure C.4 shows the posterior samples of coefficients associated with the magnitude $\beta_{C-o}$ and half-life of the carry-over effects ${hl}_{C}$ from previous testing sessions. The posterior samples of $\beta_{C-o}$ (the left panels) are all negative which suggest that a beneficial carry-over effect exists for both tasks. The posterior samples of half-lives show that the half-life of the carry-over effect is around 4 days for the Pro-point task and around 40 days for the Anti-point task. Put together, the posterior samples of half-life and magnitude of the carry-over effects suggest that there is a positive carry-over effect which attenuates much faster in Pro-point task (mean of half-life posteriors around 4 days) compared to the Anti-point task (mean of half-life posteriors around 42 days).


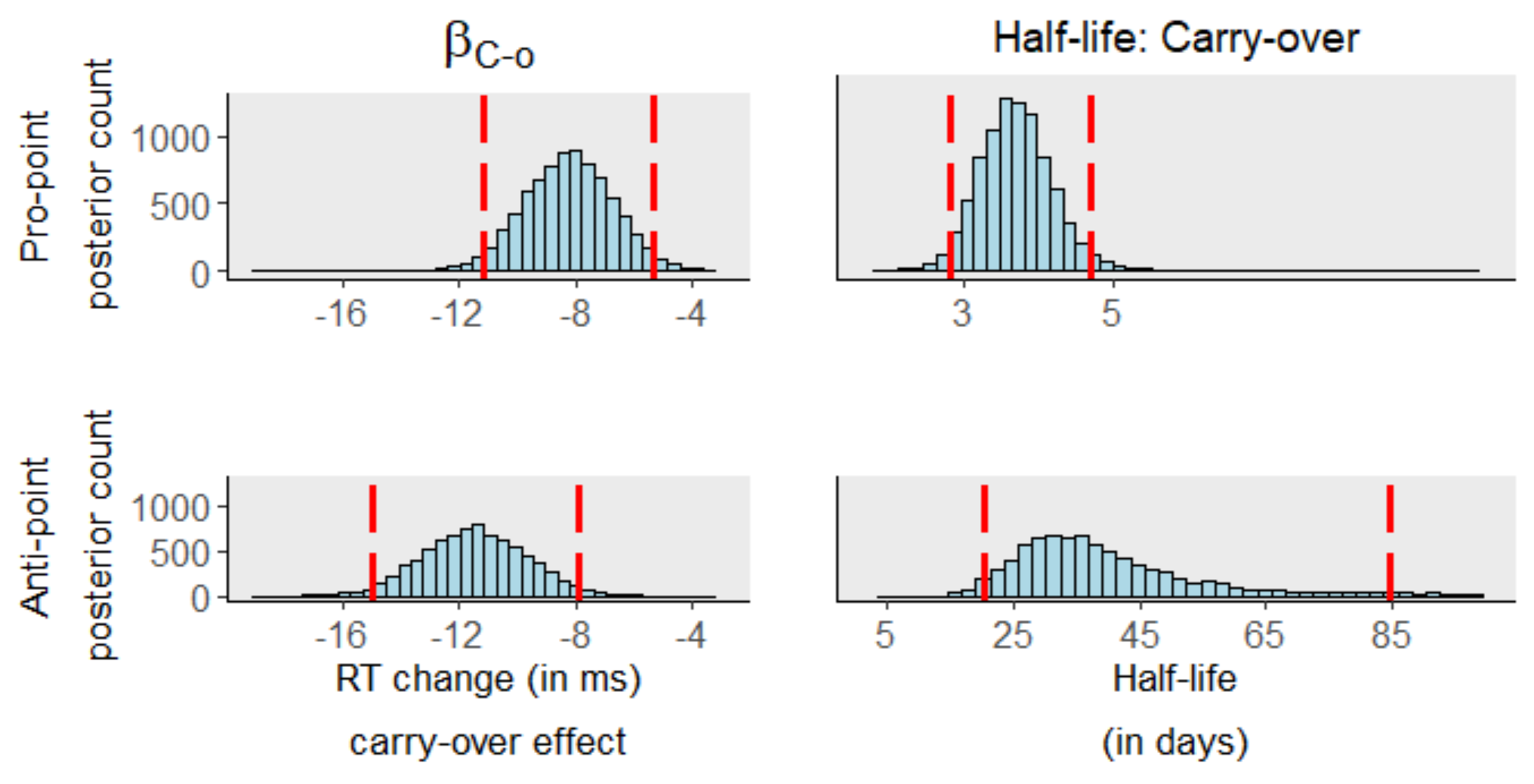


Figure C.4. Histogram of coefficients related to the carry-over effect. The negative posterior samples of $\beta_{C-o}$ (left panels) show that beneficial carry-over effects exists for both tasks. The posterior samples of the half-life of the carry-over effects (the right panels) show that carry-over benefits attenuate relatively fast for the Pro-point task compared to the Anti-point task. The dashed red lines show the 95% credible interval.

Figure C.5 shows the histograms of the gap between successive sessions for control and soccer groups and shows that the gap is on average bigger for the control group. In other words, the control group on average benefited less from the positive effects of small gaps between consecutive sessions. This is especially the case for the Pro-point task where the positive carry-over effects dissipate quickly (half-life of around four days).


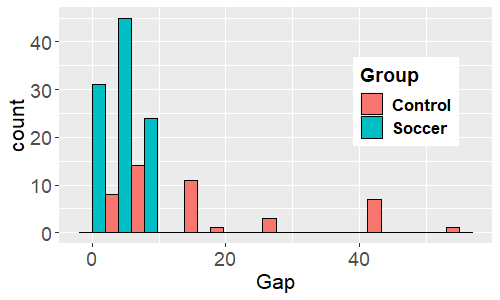


Figure C.5. Histogram of the gap between consecutive sessions for control and soccer groups. The histogram shows that there are distribution differences between groups in the data with the control group having greater time intervals between testing sessions than did the soccer players. As a result, soccer players took more advantage from small gaps between sessions (i.e., faster RT) when performing the task than control athletes.

#### Exercise, Age and Group effects

Figure C.6 shows the posterior distribution of the remaining population level effects ($\beta_{Pre/post}$, $\beta_{Age}$ and $\beta_{Group}$). The posterior samples of $\beta_{Pre/post}$ (the left panels) show that athletes are on average around 13 ms faster on the Pro-point task and around 19 ms faster at the Anti-point task immediately after exercise. The posterior samples of $\beta_{Age}$ (the middle panels) suggest that in both tasks, the higher the age, the faster the RTs, and on average, being a year older makes an athlete around 15 ms faster. Note that all the participants in this study are between the ages of thirteen and nineteen and therefore the results are consistent with Li et al.,^1^ which showed (using the same tablet-based tasks) a nonlinear U-shaped effect of age on RT on both tasks with the RTs becoming faster until the age of early to mid-twenties and getting slower afterwards. Figure C.7 shows the histogram of $age$ covariate for control and soccer groups and shows that the soccer players were older than the control group and therefore their RT on average was affected more positively by the age factor compared to the control group. Finally, the result of $\beta_{Group}$ (Figure C.6, right panel) suggests that the posterior samples are more or less centered around zero, which suggests that the differences in average RTs between soccer players and the control group were due to differences that are included in the model (i.e., time related factors of testing and factors related to the head impacts).


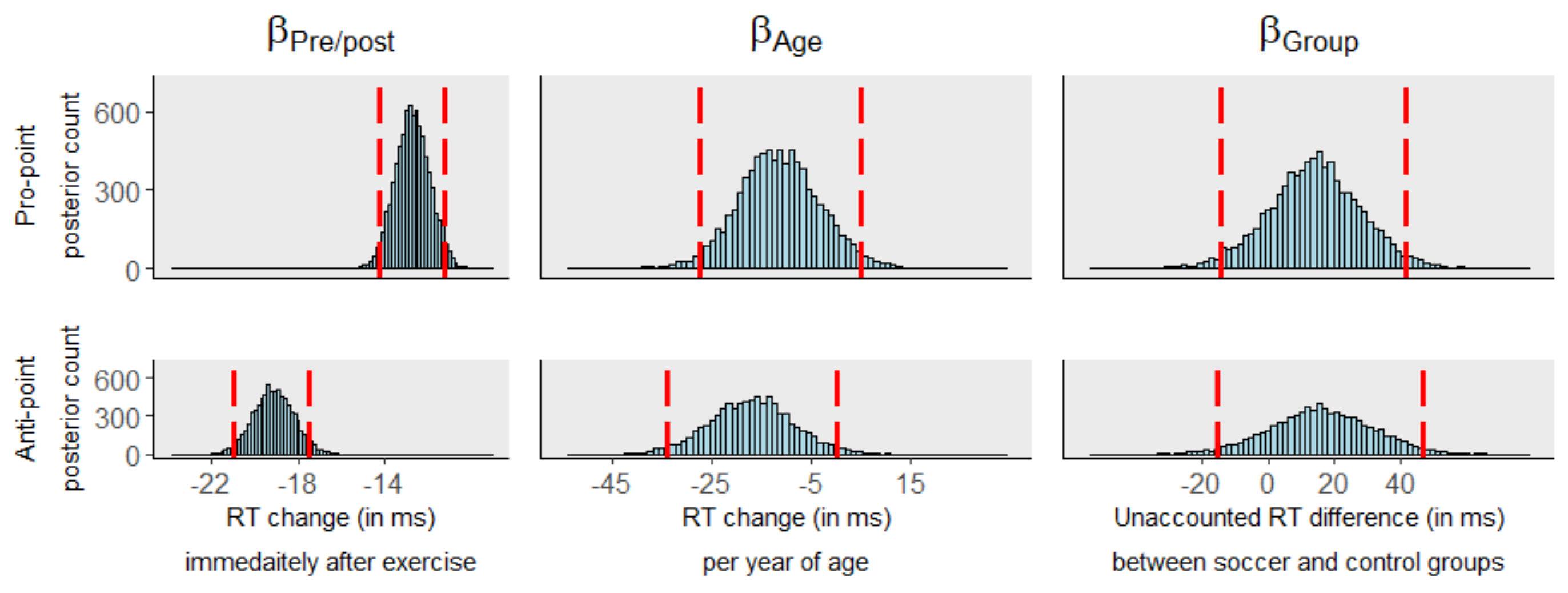


Figure C.6. Histogram of posterior samples of $\beta_{Pre/post}$, $\beta_{Age}$ and $\beta_{Group}$. Posterior samples of $\beta_{Pre/post}$ (the left panels) show the magnitude of the immediate effects of physical exercise. Posterior samples of $\beta_{Age}$ (the middle panels) show the older teenagers on average are faster on both tasks. Additionally, $\beta_{Group}$(the right panels) suggests that there is no unaccounted RT difference between soccer players and the control group in either Pro-point or Anti-point task. The dashed red lines show the 95% credible interval.


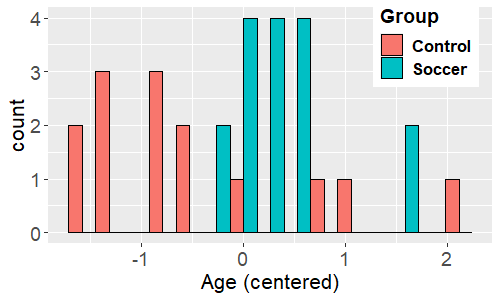


Figure C.7. Histogram of $age$ covariate (centered) for control and soccer groups. The histogram shows that there are distribution differences between groups in the data with the soccer players being on average older, which is associated with faster RTs in this age range in both Pro-point and Anti-point tasks.

# Reference

# Li EK, Lee S, Patel SS, et al. Age-dependent Performance on Pro-point and Anti-point Tasks. Front Psychol 2018;9:2519; doi: 10.3389/fpsyg.2018.02519.
