# Supplementary material for: Quantifying the Magnitude and Longevity of the Effect of Repetitive Head Impacts in Adolescent Soccer Players: Deleterious Effect of Long Headers Extend Beyond a Month
Source: Neurotrauma Rep. 2023 Apr 21;4(1):267–75. doi: 10.1089/neur.2022.0085 (PMC10122256; doi:10.1089/neur.2022.0085)
Supplement: Supplemental data [file Suppl_AppendixA.docx]

# Appendix A

# Model description

Equation (A.1) shows the structure of the model. RTs are assumed to be normally distributed with mean $\mu$ and standard deviation $\sigma_{e}$. $\beta_{1}$ denotes the population level intercept, $u\left[ i \right]$denotes the athlete level intercept (i.e., a random effect in the context of mixed effect modeling). There are four parameters in the model related to the effect of short and long headers: Two parameters modeling the magnitude of the head impacts on RT ($\beta_{Short}$ and $\beta_{Long}$ for short and long headers respectively) and two parameters modeling the half-life associated with the effect of each short and long headers (${hl}_{S}$ and ${hl}_{L}$,respectively). ${hl}_{S}$ and ${hl}_{L}$ are embedded in $f_{Short}({{hl}_{S})}_{i,m}$ and $f_{Long}({{hl}_{L})}_{i,m}$, which are weighted sums of each type of headers for the $m^{th}$ session of $i^{th}$athlete. Weight of each header depends on the number of days between the head impact and testing day. There are also four parameters related to the time-related effects of testing: $\beta_{Practice}$, $\beta_{Days}$, $\beta_{C-o}$ and ${hl}_{C}$. $\beta_{Practice}$ estimates the magnitude of the effect of prior practice sessions on RT (denoted as $t_{p}$) and $\beta_{Days}$ estimates the magnitude of the effect of the number of days since the participant’s first RT task session (to take into account the possibility of developmental changes across time in these adolescent youth athletes, denoted as $t_{d}$). $\beta_{C-o}$ and ${hl}_{C}$ are parameters that model the magnitude and half-life of the carry-over effects of prior testing session. ${hl}_{C}$ is embedded in $f({hl}_{C})$. In addition to coefficients corresponding to the time related factors of head impacts and testing, there are three other parameters: $\beta_{Pre/post}$, $\beta_{Age}$ and $\beta_{Group}$. $\beta_{Pre/post}$ models the immediate benefits of physical exercise, which was possible because athletes in this study were tested before and after each training session. $\beta_{Age}$ models the effect of athletes’ age in their first session (the covariate $age$ is deviation from average age in years) and $\beta_{Group}$corresponds to a categorical variable in the model differentiating between “Soccer” and “Control” participants. $I$ is the indicator function, and therefore, $I_{Post}$equals one for RTs of “Post” session and zero for RTs of the “Pre” session and similarly, $I_{Soccer}$ is one for RTs of soccer players and zero for RTs of the other participants.

|  | $RT\sim Normal\left( \mu,\sigma_{e} \right)$  Where for $i^{th}$athlete and $m^{th}$ session:  $\mu_{i,m}=\boldsymbol{\beta}_{\boldsymbol{1}}+u\left[ i \right]+\boldsymbol{\beta}_{\boldsymbol{Short}}\times f_{Short}({\boldsymbol{hl}_{\boldsymbol{S}})}_{i,m}+$ $\boldsymbol{\beta}_{\boldsymbol{Long}}\times f_{Long}({\boldsymbol{hl}_{\boldsymbol{L}})}_{i,m}+$ $\boldsymbol{\beta}_{\boldsymbol{Practice}}\times t_{p}+\boldsymbol{\beta}_{\boldsymbol{Days}}\times t_{d}+$  $\boldsymbol{\beta}_{\boldsymbol{C-o}}\times{f(\boldsymbol{hl}_{\boldsymbol{C}})}_{m}+\boldsymbol{\beta}_{\boldsymbol{Pre/post}}\times I_{Post}+ \boldsymbol{\beta}_{\boldsymbol{Age}}\times age+\boldsymbol{\beta}_{\boldsymbol{Group}}\times I_{Soccer}$ | (A.1) |
| --- | --- | --- |

The proposed model is a nonlinear regression because the half-life related parameters are in the denominator of $f_{Short}({hl}_{S})$, $f_{Long}({hl}_{L})$ and $f({hl}_{C})$. The parameters of the model are estimated using Bayesian methods.

# Time related factors of head impacts: accumulation with attenuation

We assumed that the effect of a RHIs on RT is maximal a day after the hit occurs^1^ and it attenuates as time goes by according to a Gaussian decay, and that short and long headers have different decay parameters ($s_{S}$ and $s_{L}$ respectively). Equation (A.2) shows the attenuating factor of session $j$’s short headers on session $m$’s RTs ($Decay\left( {Gap}_{j,m} \right)$) given that session$m$ happened after session $j$. ${Gap}_{j,m}$is the number of days between the testing sessions $j$ and$m$. $s_{S}$ is a parameter to be estimated and relates to the half-life of the attenuation factor according to Equation (A.3). The equations are similar for long headers.

| $Decay\left( {Gap}_{j,m} \right)=\exp\left( \frac{-\left( {Gap}_{j,m} \right)^{2}}{2\times{s_{S}}^{2}} \right)$ | (A.2) |
| --- | --- |
| ${Half life}_{Short headers}=\sqrt{{s_{S}}^{2}\times2\log\left( 2 \right)}$ | (A.3) |

Finally, $f_{Short}({hl}_{S})$ and $f_{Long}({hl}_{L})$ are the weighted sums of all the short and long headers, respectively, received prior to a testing session where ${hl}_{S}$ and ${hl}_{L}$ are half-lives of the effect of short and long headers respectively. Equation (A.4) shows the $f_{Short}({hl}_{S})$ for $i^{th}$athlete and $m^{th}$testing session and Equation (A.5) shows a more concise notation. In Equations (A.4) and (A.5), $N_{i,j}\left( Short \right)$denotes the number of recorded short headers for$i^{th}$ athlete on his $j^{th}$ session. Equations for $f_{Long}({hl}_{L})$ are similar (not written to avoid repetition).

|  | ${f_{Short}({hl}_{S})}_{i,m}=\sum_{j=1}^{m-1} \exp\left( \frac{-\left( {Gap}_{j,m} \right)^{2}}{2\times s_{S}^{2}} \right)\times N_{i,j}(Short)$ | (A.4) |
| --- | --- | --- |
|  | ${f_{Short}({hl}_{S})}_{i,m}=\sum_{j=1}^{m-1} \mathrm{Decay} ({Gap}_{j,m})\times N_{i,j}(Short)$ | (A.5) |

As an example, Equation (A.6) shows the$f_{Short}({hl}_{S})$ for the 3^rd^ session of $i^{th}$ athlete who has eight and eleven short headers received seven and four days prior to the 3^rd^ session, respectively.

|  | ${f_{Short}({hl}_{S})}_{i,3}=$ $\exp\left( \frac{-\left( 7 \right)^{2}}{2\times s_{S}^{2}} \right)\times8+\exp\left( \frac{-\left( 4 \right)^{2}}{2\times s_{S}^{2}} \right)\times11$ | (A.6) |
| --- | --- | --- |

# Modeling the carry-over effects of prior testing session

One of the time-related factors of testing considered in our study is the number of days since the previous training session (denoted as ${Gap}_{m-1,m}$ for the m^th^ session). $Gap$ covariate is unbalanced between soccer players and control group (with control group having bigger gaps between testing sessions) and if there is a carry-over effect (due to recency effects of previous testing or physical exercise of previous training session), then it matters how long ago the previous session occurred. Therefore, if there exist any carry-over effects across sessions and given that there is an unbalance in the $Gap$ covariate between soccer and control athletes, not including the $Gap$ covariate can affect the estimate of the effect of head impacts. The prior session’s carry-over effect, if it exists, is assumed to be maximum a day after and attenuates over time. The attenuation of the effect is modeled similar to the way that attenuation of the effect of head impacts is modeled. Equation (A.7) shows the covariate that takes into account the gap effect for the $m^{th}$testing session.

| ${f(C-o)}_{m}=\exp\left( \frac{-\left( {Gap}_{m-1,m} \right)^{2}}{2\times{s_{C}}^{2}} \right)$ | (A.7) |
| --- | --- |

${Gap}_{m-1,m}$is the number of days since the previous testing session and $s_{C}$ is a parameter to be estimated and relates to the half-life of the attenuation factor of the session carry-over effect.

Table A.1.

*Prior specification, 95% credible intervals, posterior estimate, and corresponding* $\hat{R}$ *for population level parameters.*

|  |  | **Label** | **Prior Specification** | **Estimate**  **(mean, ms)** | **95% Credible Interval (ms)** | $\hat{\boldsymbol{R}}$ |
| --- | --- | --- | --- | --- | --- | --- |
| $\beta_{1}$ | Pro-point | Intercept | Normal(442,100) | 450.64 | (430.42,471.46) | 1 |
|  | Anti-point |  | Normal(546,100) | 540.43 | (518.67,562.87) | 1 |
| $\beta_{Short}$ | Pro-point | Short headers | Normal(0,100) | -1.1 | (-1.31,-0.91) | 1 |
|  | Anti-point |  |  | -1.17 | (-1.39,-0.95) | 1 |
| ${hl}_{S}$ | Pro-point | Half-life (short headers) | <500 days | 5.19 | (4.27,6.09) | 1 |
|  | Anti-point |  |  | 6.51 | (5.02,8.15) | 1 |
| $\beta_{Long}$ | Pro-point | Long headers | Normal(0,100) | 3.69 | (3.2,4.17) | 1 |
|  | Anti-point |  |  | 3.2 | (2.64,3.77) | 1 |
| ${hl}_{L}$ | Pro-point | Half-life (long headers) | <500 days | 309.07 | (13.76,491.01) | 1 |
|  | Anti-point |  |  | 358.08 | (100.49,494.09) | 1 |
| $\beta_{Practice}$ | Pro-point | Practice Effect | Normal(0,100) | -5.21 | (-5.99,-4.41) | 1 |
|  | Anti-point |  |  | -7.34 | (-8.33,-6.37) | 1 |
| $\beta_{Days}$ | Pro-point | Developmental Effects | Normal(0,100) | -0.23 | (-0.29,-0.17) | 1 |
|  | Anti-point |  |  | -0.34 | (-0.41,-0.26) | 1 |
| $\beta_{C-o}$ | Pro-point | Carry-over from previous session | Normal(0, 100) | -8.23 | (-11.16,-5.31) | 1 |
|  | Anti-point |  |  | -11.47 | (-14.98,-7.9) | 1 |
| ${hl}_{C}$ | Pro-point | Half-life (carry-over) | <100 days | 3.72 | (2.81,4.7) | 1 |
|  | Anti-point |  |  | 42.8 | (20.3,84.59) | 1 |
| $\beta_{Pre/post}$ | Pro-point | Immediate exercise | Normal(0,100) | -12.75 | (-14.3,-11.2) | 1 |
|  | Anti-point |  |  | -19.16 | (-20.95,-17.33) | 1 |
| $\beta_{Age}$ | Pro-point | Age (centered) | Normal(0, 100) | -11.6 | (-27.59,4.79) | 1 |
|  | Anti-point |  |  | -17.1 | (-34,0.14) | 1 |
| $\beta_{Group}$ | Pro-point | Soccer vs Control | Normal(0, 100) | 13.86 | (-14.41,41.73) | 1 |
|  | Anti-point |  |  | 16.04 | (-15.53,46.89) | 1 |

# Reference

# Balagopal R, Won M, Patel SS, et al. Heading-related slowing by twenty-four hours in youth athletes. J Neurotrauma 2020;37(24):2664-2673.
