# Supplementary material for: Quantifying the Magnitude and Longevity of the Effect of Repetitive Head Impacts in Adolescent Soccer Players: Deleterious Effect of Long Headers Extend Beyond a Month
Source: Neurotrauma Rep. 2023 Apr 21;4(1):267–75. doi: 10.1089/neur.2022.0085 (PMC10122256; doi:10.1089/neur.2022.0085)
Supplement: Supplemental data [file Suppl_AppendixB.docx]

# Appendix B

# The inference method

There are at least two approaches to do statistical inference: A Null Hypothesis Significance Testing (NHST) approach and a Bayesian approach. In a NHST approach the parameter is assumed to be a fixed number and is tested against a hypothesis of no effect. For example consider $\beta_{Short}$. If we were to use a NHST approach, we could only claim whether there is evidence to reject the null hypothesis of $\beta_{Short}=0$ or not. It is not possible to answer questions such as what is the probability of $\beta_{Short}$ being bigger than zero, or being close to zero, etc. On the other hand in a Bayesian approach, it is possible to compute the probability of$\beta_{Short}>0$, or $-0.01<\beta_{Short}<0.01$, or any other probability because parameters are treated as random variables. That is, it is possible to describe a parameter’s properties only using a Bayesian inference approach. As a consequence, in a Bayesian approach it is possible to quantify uncertainty which can be especially important when dealing with observational data.

Bayesian statistics uses Bayes theorem to calculate the posterior distribution, $P\left( Ɵ | data \right)$ (Equation (C.1)):

|  | $P\left( Ɵ \vert data \right)= \frac{P\left( data \vertƟ \right)\times P(Ɵ)}{P(data)}$ | (B.1) |
| --- | --- | --- |

In Equation (B.1), $Ɵ$ denotes the vector of all parameters, $P\left( data | Ɵ \right)$ is called the likelihood and $P(Ɵ)$ is called the prior. Note that there is no $Ɵ$ term in the denominator $P(data)$ and therefore the posterior is proportional to prior multiplied by likelihood. The nonlinear regression model equations (Equation (A.1)) give the likelihood, but the prior should be specified. One advantage of Bayesian statistics is the possibility of incorporating previous knowledge into the model by specifying the prior distribution. A prior distribution specification can vary from flat/uniform (uninformative) to super vague, weakly informative, or informative.^1^ Also note that for some parameters in the model there are natural constraints. For example, the parameters signifying standard deviations or a half-life must be positive. The prior distributions and the constraints for the parameters in the model are shown in Table A.1. Except for the intercept ($\beta_{1}$) all the priors are the same for Pro-point and Anti-point tasks. No prior specification means a flat prior was assumed. In data-driven studies, where the goal is to “let the data speak for themselves,” the priors are defined either flat or super vague. However, due to problems that may arise due to flat or super vague priors,^2^ our goal was to inform priors for parameters where there exists some a priori knowledge. For $\beta_{1}$, a weakly informative prior was defined relying on a previous study conducted using the same testing device (the tablet) and the same age group.^3^ Additionally, the parameter that is related to the half-life of short and long heading effects on RT (${s_{S}}^{2}$ and ${s_{L}}^{2}$) was defined to correspond to half-lives between 0 and 500 days, which matched the timeline of the experiment (in the soccer group, group with headers, the maximum number of days between first session and last session of testing was 29 days), and the half-life of the carry-over effects from the previous testing session was restricted to be between 0 and 100. There was no prior knowledge on other population level parameters and therefore a super vague prior was defined for all of them. There was no knowledge on parameters signifying standard deviations ($\sigma_{e}$and$\sigma_{\mathrm{sj}}$), so the only constraint was to limit them to be positive.

Note that having both prior and likelihood is not enough to identify the posterior distribution; and, unless the posterior distribution ends up corresponding to a known probability density function (e.g., a normal distribution), an algorithm is needed to sample from the posterior. The distribution of samples gives a sense what the probability distribution of the posterior looks like. We used RStan to sample the posterior of our model. Details of the algorithms used in RStan can be found in Carpenter et al.^4^ In order to make sure that the posterior samples are converging regardless of the starting point of the iterative sampling, four independent chains with different starting points were run, each for 6000 iterations (i.e., 6000 samples) with the first 4000 samples as warm-up. Convergence of the four chains to a common distribution was confirmed using the Gelman-Rubin ($\hat{R}$) statistic.^5^

# References

# Gelman A, Carlin JB, Stern HS, Dunson DB, Vehtari, A, Rubin, DB. *Bayesian Data Analysis*. London (UK): Chapman and Hall/CRC; 2013.

# Seaman III JW, Seaman Jr JW, Stamey JD. Hidden dangers of specifying noninformative priors. Am Stat 2012;66(2):77-84

# Zhang MR, Red SD, Lin AH, et al. Evidence of cognitive dysfunction after soccer playing with ball heading using a novel tablet-based approach. PloS One 2013;8(2).

# Carpenter B, Gelman A, Hoffman MD, et al. Stan: A probabilistic programming language. J Stat Softw 2017;76(1):1-32.

# Gelman A, Rubin DB. Inference from iterative simulation using multiple sequences. Stat Sci 1992;7(4):457-472.
